# Cortactin Phosphorylated by ERK1/2 Localizes to Sites of Dynamic Actin Regulation and Is Required for Carcinoma Lamellipodia Persistence

**DOI:** 10.1371/journal.pone.0013847

**Published:** 2010-11-04

**Authors:** Laura C. Kelley, Karen E. Hayes, Amanda Gatesman Ammer, Karen H. Martin, Scott A. Weed

**Affiliations:** Department of Neurobiology and Anatomy, Program in Cancer Cell Biology, Mary Babb Randolph Cancer Center, West Virginia University, Morgantown, West Virginia, United States of America; Dresden University of Technology, Germany

## Abstract

**Background:**

Tumor cell motility and invasion is governed by dynamic regulation of the cortical actin cytoskeleton. The actin-binding protein cortactin is commonly upregulated in multiple cancer types and is associated with increased cell migration. Cortactin regulates actin nucleation through the actin related protein (Arp)2/3 complex and stabilizes the cortical actin cytoskeleton. Cortactin is regulated by multiple phosphorylation events, including phosphorylation of S405 and S418 by extracellular regulated kinases (ERK)1/2. ERK1/2 phosphorylation of cortactin has emerged as an important positive regulatory modification, enabling cortactin to bind and activate the Arp2/3 regulator neuronal Wiskott-Aldrich syndrome protein (N-WASp), promoting actin polymerization and enhancing tumor cell movement.

**Methodology/Principal Findings:**

In this report we have developed phosphorylation-specific antibodies against phosphorylated cortactin S405 and S418 to analyze the subcellular localization of this cortactin form in tumor cells and patient samples by microscopy. We evaluated the interplay between cortactin S405 and S418 phosphorylation with cortactin tyrosine phosphorylation in regulating cortactin conformational forms by Western blotting. Cortactin is simultaneously phosphorylated at S405/418 and Y421 in tumor cells, and through the use of point mutant constructs we determined that serine and tyrosine phosphorylation events lack any co-dependency. Expression of S405/418 phosphorylation-null constructs impaired carcinoma motility and adhesion, and also inhibited lamellipodia persistence monitored by live cell imaging.

**Conclusions/Significance:**

Cortactin phosphorylated at S405/418 is localized to sites of dynamic actin assembly in tumor cells. Concurrent phosphorylation of cortactin by ERK1/2 and tyrosine kinases enables cells with the ability to regulate actin dynamics through N-WASp and other effector proteins by synchronizing upstream regulatory pathways, confirming cortactin as an important integration point in actin-based signal transduction. Reduced lamellipodia persistence in cells with S405/418A expression identifies an essential motility-based process reliant on ERK1/2 signaling, providing additional understanding as to how this pathway impacts tumor cell migration.

## Introduction

Tumor cell motility and invasion is a central problem in cancer that is paramount in contributing to metastasis [1]. Tumor cells move through successive series of coordinated and integrated stages, with formation of protrusive membranous structures including filopodia, invadopodia and lamellipodia required for initiation and maintenance of invasion and migration [2], [3], [4], [5]. Central to the movement of most carcinoma cell types undergoing single or collective migration is the production of lamellipodia at the leading edge of the cell. Lamellipodia are planar protrusive extensions of the plasma membrane produced by motile cells in two- and three-dimensional settings [6]. Lamellipodia extension drives cell migration through integrin-based adhesion with the underlying substratum, providing the necessary traction for contractile-based translocation of the cell body to generate productive movement [7]. It is generally accepted that dynamic regulation of the cortical actin cytoskeleton through cycles of actin polymerization and depolymerization are responsible for generating the propulsive force needed for lamellipodia extension [8].

The actin binding protein cortactin is a major component of lamellipodia that regulates the lamellipodia actin network through several pro-migratory signaling pathways [9], [10], [11]. Biochemical analysis indicates that cortactin interacts directly with the actin-related (Arp) 2/3 complex through a conserved acidic motif within the amino terminus, initiating Arp2/3-dependent actin nucleation responsible for lamellipodia formation [12], [13], [14]. Simultaneous binding of cortactin to Arp2/3 complex and the resulting filamentous (F)-actin dendritic network serves to stabilize F-actin branchpoints [13], while binding of the cortactin carboxyl-terminal Src homology (SH)3 domain to the Arp2/3 activator N-WASp or the N-WASp scaffolding protein WIP additionally promotes Arp2/3 activation and cell motility [15], [16], [17].

Although the biochemical features of cortactin seem to point to a straightforward role in lamellipodia actin regulation, studies of cortactin function in lamellipodia have proven controversial, suggesting to a more complex role in cell migration. RNA interference studies have yielded conflicting results in regards to lamellipodia dynamics, with cortactin knockdown resulting in decreased lamellipodia stability and reduced persistence consistent with a role in lamellipodia actin network stabilization [18], [19], [20]. However, similar studies in different cell types suggest cortactin downregulation increases the length of extending lamellipodia [21]. Furthermore, recent analysis of lamellipodia dynamics in cortactin^−/−^ fibroblasts indicates that cortactin does not play a role in directly regulating lamellipodia protrusion or Arp2/3-based actin dynamics, but rather is important in mediating upstream activation of the small GTPases Rac1 and Cdc42, which in turn regulate WAVE2 and N-WASp activity [22]. While these reported discrepancies regarding cortactin function in lamellipodia have yet to be fully reconciled, it is clear that cortactin is an important regulator for normal and tumor cell migration in many cell systems [11], [23]. An unambiguous role for cortactin has been shown in invadopodia, where removal of cortactin by RNA interference ablates invadopodia formation in multiple invasive tumor cell types [24], [25], [26].

Besides regulating Arp2/3-based cortical actin networks by direct interactions, cortactin also functions as a key mediator in several kinase-based signal transduction cascades that indirectly govern Arp2/3 activity and cell movement. Cortactin is a well-defined target for Src kinase [27], phosphorylating human cortactin on tyrosine residues Y421, Y470 and Y486 within the proline-rich (PR) carboxyl-terminal domain [28]. Several other receptor and cytoplasmic tyrosine kinases target these residues, including Fyn [29], Fer [30], Arg/Abl [31], c-Met [32] and Erb2 [33]. The diverse array of tyrosine kinases that phosphorylate cortactin at Y421/Y470/Y486 indicates that these sites collectively serve as a point of convergence for multiple signaling pathways. Cortactin phosphorylated at tyrosines 421, 470 and/or 486 localizes within lamellipodia [34], creating Src homology (SH)2 docking sites that facilitate binding of tyrosine kinases and adaptor proteins indirectly responsible for regulating cortical actin dynamics and subsequent cell movement through N-WASp-mediated Arp2/3 activity [35], [36], [37].

Besides tyrosine phosphorylation, cortactin is a target for multiple serine/threonine kinases [38]. Stimulation of tumor cells with epidermal growth factor (EGF) leads to phosphorylation of serine residues 405 and 418 within the PR domain, coincident with a characteristic shift in cortactin electrophoretic mobility from 80 kDa to 85 kDa in SDS-PAGE [39], [40]. The mobility shift and phosphorylation of S405/S418 are impaired by pharmacologic inhibition of mitogen activated protein/extracellular signal regulated kinase kinase (MEK)1/2, and biochemical evidence indicates that the MEK effector kinases ERK1/2 directly phosphorylate cortactin at these sites [40]. Phosphorylation of S405/S418 enhances binding of the cortactin SH3 domain to N-WASp, indicating a functional role in stimulating Arp2/3-mediated actin dynamics independent of tyrosine phosphorylation [15]. This is supported by studies expressing phosphorylation-null and phosphomimetic point mutant constructs in cells, suggesting that S405/S418 phosphorylation plays a critical role in regulating cellular actin polymerization necessary to promote cell migration [41] and invadopodia function [26]. In addition, p21 activated kinase 1 (PAK1) phosphorylates cortactin at S405/S418, serving to stimulate N-WASp activity required for clathrin-independent endocytosis [42]. While studies to date implicate cortactin S405/418 phosphorylation in promoting N-WASp-mediated Arp2/3 actin structures, the subcellular localization of phosphorylated S405/418 cortactin, as well as the precise role S405/418 phosphorylation plays in regulating lamellipodia dynamics have not been evaluated.

In this study, we have generated site-specific antibodies against phosphorylated cortactin S405 and S418 to determine the spatial and temporal localization of cortactin in dynamic actin structures and human tumors, and to evaluate signaling interplay between cortactin tyrosine and serine phosphorylation events. We also determined the effects of S405/418 cortactin phosphorylation on EGF-induced cell migration, adhesion and lamellipodia dynamics in carcinoma cells.

## Methods

### DNA Constructs and siRNA

For Myc-tagged human cortactin expression constructs, the wild-type human cortactin cDNA subcloned into pcDNA FLAG2AB [43] was used as a template for producing point mutants by site-directed mutagenesis (QuickChange; Stratagene, La Jolla, CA). Codon alterations in human cortactin were: S405A, S418A, S405A/S418A, Y421F, Y470F, Y486F, Y421F/Y470F/Y486F and W492K. Cortactin cDNAs were amplified by PCR as BamHI-EcoRI fragments and subcloned into BamHI-EcoRI digested pRK5Myc [44]. Murine GFP-tagged expression constructs were produced using pcDNA3FLAG2AB wild-type murine cortactin [12] as the template for mutagenesis, then subcloned as EcoRI-KpnI PCR fragments into pAcGFP-C1 (Clontech, Mountain View, CA). The temperature-sensitive vSrc LA29 construct was previously described [45]. mCherry-β-actin was obtained from D. Schafer (University of Virginia), with the parent construct produced by R. Tsien (University of California, San Diego). Small interfering (si)RNA targeting rodent cortactin (5′-GCTTCGAGAGAATGTCTTC-3′) was purchased from Thermo Scientific (Waltham, MA).

### Cell lines and Transfection

The HNSCC cell lines 1483 [46], UMSCC1 and UMSCC2 [47] were maintained as described [48]. SYF cells were obtained from the American Type Culture Collection (Manassas, VA) and maintained according to the supplied protocol. The rat mammary adneocarcinoma line MTLn3 was maintained in alpha-MEM supplemented with 10% fetal bovine serum, 1% L-glutamine and 1% penicillin-streptomycin. Transient transfections were conducted with 3×10^6^ cells and two micrograms of plasmid construct or siRNA using the Nucleofector I device (Amaxa Biosystems, Berlin, Germany).

### Antibodies

**Figure 1:**
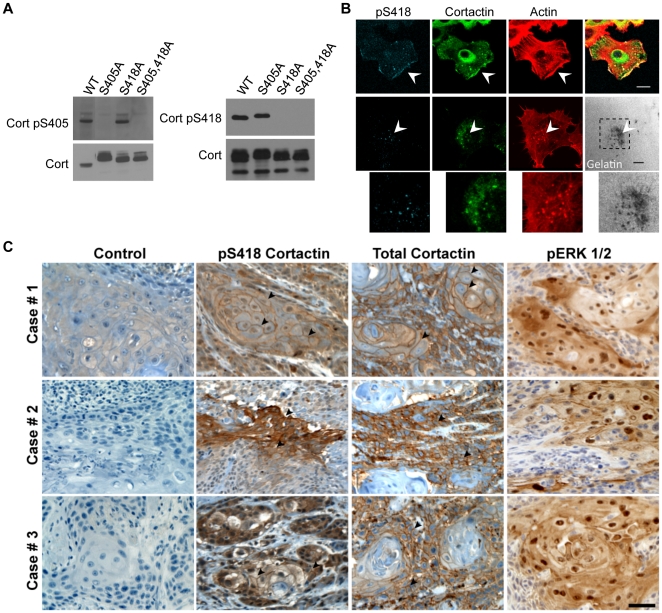
Specificity and validation of pS405 and pS418 phospho-specific cortactin antibodies. (**A**) Phospho-specific recognition of anti-cortactin pS405 and pS418 antibodies. Clarified lysates (50 micrograms) from 1483 cells transfected with Myc-tagged wild-type cortactin (WT), Myc-cortactin S405A, Myc-cortactin S418A or Myc-cortactin S405A,S418A point mutants were immunoblotted with affinity purified anti-Cort-pS418 (left) and anti-Cort-pS405 (right) antibodies. (**B**) Localization of pS418 cortactin in areas of motile and invasive actin dynamics. UMSCC2 cells (*top row*) were serum starved for 16 h prior to stimulation with 100 nanograms/ml EGF for 1 h to induce lamellipodia formation, while UMSCC1 cells (*middle row*) were plated on FITC-conjugated gelatin coated coverslips (pseudocolored white) for 6 h to promote invadopodia formation. Cells were fixed, permeablized, and labeled with TRITC-phalloidin (Actin), anti-cortactin (Cort) and anti-cortactin-pS418 antibodies. Arrows denote localization of pS418 cortactin with total cortactin and F-actin in lamellipodia (*top*) and to invadopodia (*middle*) coinciding with areas of active matrix degradation. Bottom panels are magnified views of the indicated cellular region. Bars, 10 micrometers. (**C**) Localization of pS418 cortactin in HNSCC tumor tissue. Serial sections from three different invasive HNSCC cases were processed for immunohistochemistry with control IgG (Control), pS418 cortactin, total cortactin and phospho-ERK1/2 (pERK) antibodies. Sections were counterstained with hematoxylin. Arrowheads indicate areas of peripheral pS418 cortactin and total cortactin enrichment within each tumor sample. Bar, 100 micrometers.

Antibodies against phosphorylated serine 405 (pS405) and serine 418 (pS418) of human cortactin were produced by 21^st^ Century Biochemicals (Marlboro, MA). Synthetic phosphorylated cortactin peptides containing the sequences NH_2_-KTQTPPV[pS]PAPQPTC-COOH (cortactin pS405) and NH_2_-TEERLPS[pS]PV-COOH (cortactin pS418) were produced, conjugated to keyhole limpet cyanine and injected into rabbits. Immune serum was screened by enzyme-linked immunosorbent assay against the appropriate phosphorylated cortactin peptide coupled to bovine serum albumin. High-titer bleeds were identified for each peptide, and immune serum was passed two successive times through chromatography columns containing agarose beads coupled to the equivalent non-phosphorylated peptide. The flow through material for each peptide was subsequently passed twice through chromatography columns containing beads conjugated to the matched phosphorylated cortactin peptide. After extensive washing, bound antibodies for each phosphorylation site were eluted, concentrated and screened for specificity by Western blotting against recombinant cortactin mutant proteins harboring alanine-serine point mutations at serine 405 or 418, respectively (Fig. 1A). The anti-pS405 and anti-pS418 cortactin antibodies are currently available through Protea Biosciences (Morgantown, WV). Anti-cortactin (4F11) was used as described [48]. Anti-pY421 cortactin and anti-pY418 Src were from Invitrogen (Carlsbad, CA). Anti-ERK1/2 and pERK1/2 were from Cell Signaling (Danvers, MA). Anti-Myc epitope tag (4A6) was from Millipore (Billerica, MA). Anti-GFP (JL-8) was from Clontech (Mountain View, CA) and anti-beta-actin was from EMD4Biosciences (San Diego, CA).

### Western blotting and Immunoprecipitation

Western blotting was conducted as described [48]. Primary antibody dilutions used were: anti-pS405 cortactin (1∶4000), anti-pS418 cortactin (1∶500), anti-cortactin (1∶1000), anti-pY421 cortactin (1∶2000), anti-ERK1/2 (1∶2000), anti-pERK (1∶2000), anti-pY418 Src, anti-GFP (1∶1000) and anti-beta-actin (1∶5000). Immunoprecipitations were performed as described [34] using five micrograms of precipitating antibody captured with 40 microliters of a 50% Protein A/G bead slurry (Thermo Fisher Scientific, Pittsburgh, PA). In some cases cells were treated with selumetinib (AZD6244; ARRY-142886) or saracatinib (AZD0530) for 24 h prior to immuoprecipitation and Western blotting analysis.

### Microscopy

UMSCC2 cells were plated on fibronectin-coated coverslips (10 micrograms/ml; Sigma, St Louis, MO) and allowed to attach before serum starvation for 16 h. Cells were stimulated with 100 nanograms/ml EGF (Millipore) for 1 h before fixation. UMSCC1 cells plated on FITC-gelatin (Sigma) for 8 h were processed for confocal microscopy using Zeiss LSM 510 Meta system (Thornwood, NY) as described [43]. Anti-pS418 cortactin was used at 1∶1000, 4F11 at 1∶500 and rhodamine-conjugated phalloidin at 1∶1000 (Invitrogen, Carlsbad, CA).

For immunohistochemistry, HNSCC tissue blocks were obtained from the West Virginia University Tissue Bank and used under approval of the West Virginia University Institutional Review Board. Five-micrometer sections from formalin-fixed, paraffin-embedded blocks were processed for immunostaining using the Discovery XT automated staining system (Ventana, Tucon AZ). Briefly, after deparaffinization and antigen retrieval, sections were incubated with monoclonal rabbit anti-cortactin (Novus, Littleton, CO) at 1∶2000, anti-pS418 cortactin at 1∶25 and anti-pERK1/2 at 1∶100 dilutions. All primary antibodies were incubated in Dako diluent (Dako, Carpinteria, CA) for 1 h. Primary antibodies were detected with the Omnimap antibody horseradish peroxidase kit (Ventana). Slides were counterstained with hematoxylin and post-counterstained with bluing reagent (Ventana). Images were visualized with an Olympus AX70 microscope and captured using the MicroBrightfield system (Williston, VT).

Live cell imaging was conducted using MTLn3 cells starved for 3 h with serum-free media prior to stimulation with 100 nanograms/ml EGF. Cells were plated on delta-T4 glass bottom dishes (Fisher) coated with 10 micrograms/ml fibronectin (Sigma). Immediately following EGF addition, cells were imaged by differential interference contrast microscopy using a Nikon TE2000 inverted microscope equipped with a Roper CoolSNAP HQ charge-coupled device camera (Photometrics, Tucson, AZ). Images were captured every 5 s for 15 min (181 total frames). A Nikon LiveScan SFC swept field microscope was used for imaging cells expressing mCherry-actin using the same parameters. In all cases, GFP-cortactin expressing cells were identified by fluorescence microcopy prior to imaging. Kymograms were produced by extracting 1 pixel-width strips from each movie frame at points of initial and maximal lamellipodia extension, and assembled using ImageJ (v1.40).

### Electric Cell Substrate Impedance Sensing

To assay cell motility and adhesion, 5×10^5^ cells were plated into 8-well electric cell substrate impedance sensing dishes (ECIS; Applied Biophysics, Troy, NY). For motility measurements, cells were allowed to adhere overnight on 8W1E dishes to form a monolayer. Adhesion was assayed immediately after plating cells onto 8W10E dishes. Measurements were conducted for 24 h at 45 kHz, with reading taken at 1 min intervals. Cells treated with selumetinib were serum starved 24 h in the presence of drug prior to ECIS.

### Statistical Analysis

Differences in mean groups for migration, adhesion and kymography between control and treated groups were evaluated using one way ANOVA, followed by Student-Newman-Keuls post hoc testing. All differences were considered significant at p≤0.05. A minimum of three experimental groups were used for all analyses.

## Results

### Localization of pS418 cortactin with dynamic cortical actin structures

We developed antibodies specific to phosphoserine 405 (pS405) and phosphoserine 418 (pS418) of human cortactin to facilitate analysis of these sites. To validate antibody specificity, epitope-tagged cortactin constructs containing wild-type (WT) cortactin, cortactin with individual serine to alanine mutations at codon 405 (S405A), codon 418 (S418A) or with both codons mutated in tandem (S405,418A) were produced and transfected into 1483 cells. Total cell lysates were blotted with anti-pS405 or anti-pS418 antibodies (Figure 1A). The anti-pS405 antibody recognized the WT and S418A cortactin variants, failing to blot constructs containing the S405A mutation. Conversely, anti-pS418 blotted WT and S405A, failing to recognize cortactin constructs with S418A mutations. All cortactin variants were recognized by an anti-cortactin monoclonal antibody (Figure 1A), indicating equivalent expression of the assayed constructs. These results indicate that the anti-pS405 and anti-pS418 antibodies specifically recognize their cognate phosphorylated cortactin epitope, and that no interdependence exists between phosphorylation of cortactin S405 and S418.

To determine the subcellular localization of serine phosphorylated cortactin, we conducted indirect immunofluoresence studies on cells producing lamellipodia and invadopodia, two actin-based structures that depend in part on N-WASp activity. While the anti-pS405 antibody yielded non-specific staining in our hands (data not shown), anti-pS418 specifically labeled lamellipodia and cytoplasmic puncta (presumably vesicles) in UMSCC2 cells. In cells with a motile phenotype, anti-pS418 localized with cortactin and F-actin in these regions (Figure 1B, top row). Labeling of UMSCC1 cells plated on FITC-coated gelatin matrix with anti-pS418 indicated specific localization to subsets of invadopodia that coincided with cortactin, F-actin and areas of gelatin clearing indicative of matrix metalloproteinase mediated invadopodia activity (Figure 1B, middle and bottom rows).

In solid human tumors, cortactin and cortactin phosphorylated on tyrosine 421 (pY421) localizes to invasive tumor fronts and to cell-cell junctions [43], [48]. To determine the location of pS418 cortactin in tumor tissue, head and neck squamous cell carcinoma (HNSCC) cases were sectioned and stained with anti-pS418 (Figure 1C). Cortactin pS418 was abundant in HNSCC cell cytoplasm and was enriched in areas of cell-cell contact, displaying a pattern similar to sections labeled with a total cortactin antibody. These tumor regions also contained activated ERK1/2, as evidenced by pronounced cytoplasmic and nuclear staining of phosphorylated ERK1/2 in serial sections (Figure 1C). Enrichment of pS418 staining was not evident at margins or the invasive front in the analyzed tumors.

### Growth factor mediated phosphorylation of cortactin S405/418 is MEK dependent

**Figure 2:**
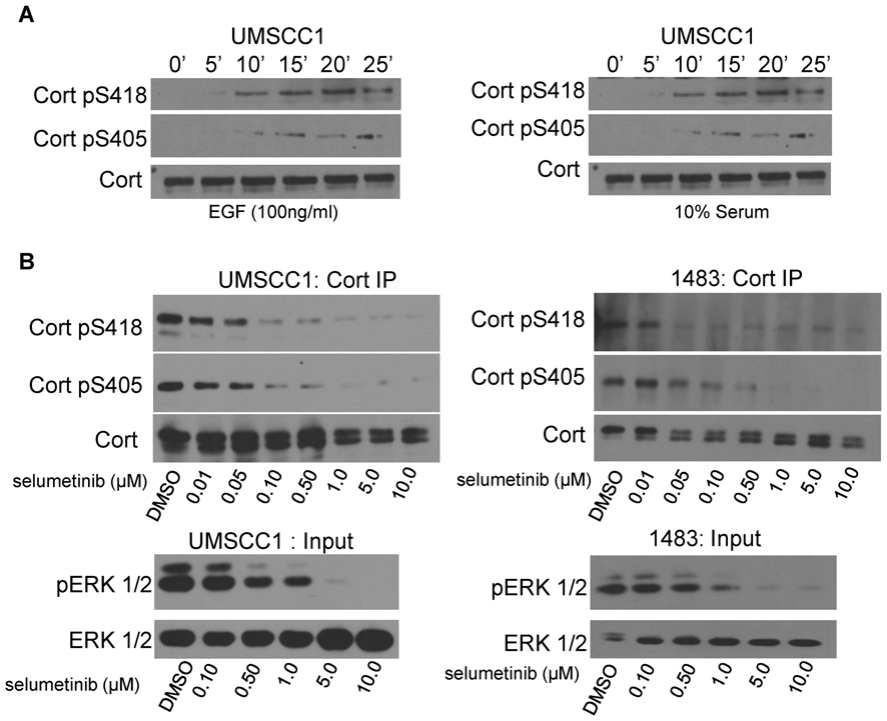
Growth factor-stimulated Erk 1/2 activation mediates phosphorylation of cortactin at serine 405 and 418. (**A**) Growth factor-induced phosphorylation of cortactin S405 and S418. Serum starved UMSCC1 cells were stimulated with EGF (left) or FBS (right) for the indicated times. Cells were lysed and analyzed by Western blotting with anti-Cort-pS418 and anti-Cort-pS405 antibodies. Blots were stripped and reprobed with a pan-cortactin antibody to confirm equal loading (*bottom*). (**B**) Pharmacologic MEK inhibition inhibits cortactin S405 and S418 phosphorylation. UMSSC1 (left) and 1483 (right) cells were serum starved in the presence of the indicated selumetinib concentrations prior to stimulation with EGF for 20 min. Cortactin immunoprecipitated from cell extracts was assayed by Western blotting with anti-Cort-pS418 and anti-Cort-pS405 antibodies. Blots were stripped and reprobed with pan-cortactin antibody as in (A) (*bottom panels*). Selumetinib efficacy was verified by the blotting of lyastes from selected timepoints with phospho-ERK1/2 (pERK1/2) and pan ERK1/2 antibodies (*bottom*). All blots are representative images from 3–4 independent experiments.

Previous biochemical work has implicated chemical inhibition of MEK and subsequent blocking of ERK1/2 activation as a major pathway responsible for cortactin S405/418 phosphorylation [40]. To further evaluate the role of the MEK-ERK1/2 pathway on cortactin phosphorylation, we utilized the anti-pS405 and pS418 cortactin antibodies to directly test the effects of MEK inhibition on cortactin pS405/418. Western blot analysis of cell extracts from EGF- and serum-stimulated UMSCC1 cells with anti-pS405 and pS418 antibodies displayed similar phosphorylation kinetics of S405 and S418, with phosphorylation of both sites first evident 10 min after stimulation (Figure 2A) and remaining phosphorylated up to 2 h (data not shown). Treatment of UMSCC1 or 1483 cells with the small molecule MEK inhibitor selumetinib [49] reduced EGF-stimulated cortactin S405/418 phosphorylation in a dose-dependent manner, where near elimination of phosphorylation at both serine residues occurred at doses ≥1 micromolar (Figure 2B). ERK1/2 activity was also reduced under similar dose conditions, although complete ablation of ERK1/2 phosphorylation was observed at doses ≥5 micromolar (Figure 2B). These data suggest that the MEK-ERK pathway is largely responsible for growth-factor induced cortactin S405/418 phosphorylation in HNSCC cells, in agreement with previous findings in other cell types [40].

### The 80 kDa to 85 kDa cortactin conformational shift is associated with serine and tyrosine phosphorylation

**Figure 3:**
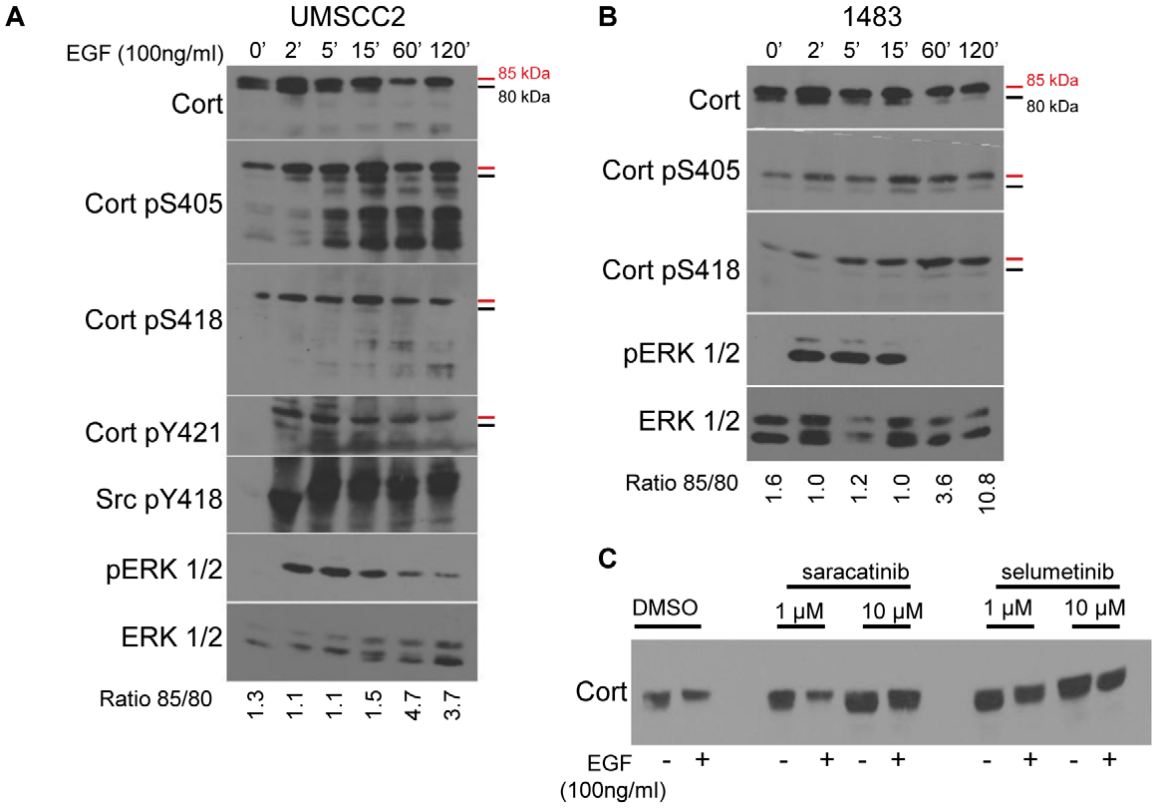
EGF-induced conversion of cortactin from 80 kDa to 85 kDa is impaired by Src and MEK1/2 inhibition. EGF induces the p80 kDa to p85 kDa shift in HNSCC cells. Serum starved UMSCC2 (**A**) and 1483 (**B**) cells were treated with 100 nanograms/ml EGF for the indicated times. Clarified lysates were assayed by Western blotting with anti-cortactin, anti-Cort-pS418, anti-cort-pS405, anti-Cort-pY421, anti-Src-pY418, anti-pErk1/2 and total Erk1/2 antibodies as indicated. Red bars denote the position of the 85 kDa cortactin form; black bars denote the 80 kDa form. The ratio of the 80 kDa and 85 kDa cortactin forms are denoted at the bottom of each set of blots. (**C**) Saracatinib and selumetinib treatment of EGF-stimulated cells inhibits the cortactin “shift”. UMSCC2 cells were treated with vehicle (DMSO), saracatinib, or selumetinib for 16 h in serum free media. Cells were stimulated with 100 nanograms/ml EGF for 1 h, lysed and analyzed by Western blot analysis with an anti-cortactin antibody.

Based on sequence analysis, the largest and most prominent cortactin isoform (cortactin “A” or “SV1”) encodes a 61.5 kDa protein [50], [51]. This cortactin form frequently migrates as an 80/85 kDa doublet in SDS-PAGE [27], [52] that has been attributed to conformational alterations within the polypeptide chain [40], [53]. Shifting from the 80 kDa to 85 kDa form is seen in response to EGF, with the resulting 85 kDa band associated with S405/418 phosphorylation [39], [40]. To directly assess the presence of pS405/418 in the two cortactin conformational isomers, serum-starved UMSCC2 (Figure 3A) and 1483 (Figure 3B) cells were stimulated with EGF and the cortactin forms in cell lysates were analyzed at successive time points with anti-pS405 and anti-pS418 antibodies. S405/418 phosphorylation was maintained in the 85 kDa cortactin form in both cell lines following serum starvation, despite of the lack of ERK1/2 activity (0 min, Figure 3A and Figure 3B). EGF stimulation resulted in complete conversion of the 80 kDa to the 85 kDa cortactin form by 1 h after EGF treatment in both cell lines, with a 3.6–4.7 fold increase in the 80 kDa/85 kDa ratio (Figure 3A and Figure 3B). Cortactin pS405 and pS418 was observed primarily in the 85 kDa form and increased at both sites during the entire time course, whereas ERK1/2 activity peaked at 15 min and rapidly declined afterwards (Figure 3A and Figure 3B). Interestingly, the phosphorylation of S405 was also associated with an increase appearance of cortactin degradation in UMSCC2 cells (Figure 3A). It is uncertain whether these products represent increased overall cortactin degradation, or if the net cortactin degradation is constant but is selectively identified by the pS405 antibody in response to EGF treatment and phosphorylation. EGF-induced Src activation and cortactin pY421 phosphorylation was sustained throughout the entire time course in UMSCC2 cells (Figure 3A), indicating that cortactin can be simultaneously phosphorylated by ERK1/2 and EGFR-stimulated tyrosine kinases. Pretreatment of UMSCC2 cells with the Src family kinase inhibitor saracatinib at 10 micromolar or selumetinib at 1 micromolar concentrations completely impaired the cortactin shift from 80 kDa to 85 kDa (Figure 3C). We assessed the specificity of these inhibitors and determined that selumetinib inhibition of MEK did not impair EGFR activity as determined by anti-pY1068 EGFR Western blotting (Figure S1A), whereas saracatinib did inhibit EGFR activation (Figure S1B) as shown previously [54]. The effects of saracatinib on blocking the EGF-mediated 80 kDa to 85 kDa cortactin conversion may therefore be due to EGFR inhibition, which in turn would inhibit activation of MEK as well as Src. The exclusive presence of pS405 and pS418 in the EGF-induced 85 kDa cortactin form, as well as the ability of MEK inhibition to impair the cortactin shift is consistent with results obtained from previous work [40].

### Cortactin serine phosphorylation *in vivo* is independent from tyrosine phosphorylation

**Figure 4:**
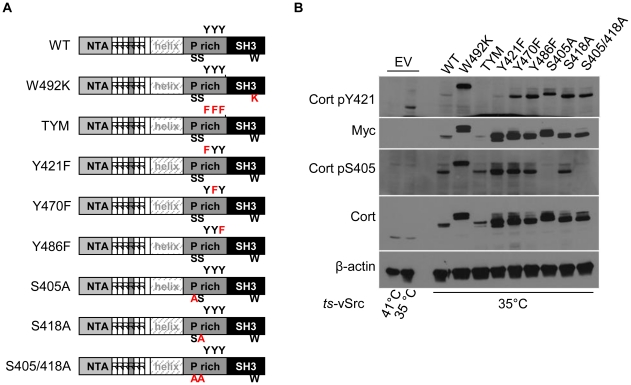
Cortactin tyrosine and serine phosphorylation resultant of v-Src activation are not interdependent. (**A**) Schematic diagram of the cortactin point mutant constructs assayed for phosphorylation. Mutated codons are denoted on the left and displayed with the corresponding mutant amino acid at the appropriate position within cortactin in red. (**B**) Murine fibroblasts lacking endogenous Src, Yes and Fyn (SYF) were transfected with murine-specific cortactin siRNA and cultured for 48 h to deplete endogenous cortactin. Cells were subsequently co-transected with the temperature-sensitive v-Src construct La29 (*ts*La29) and wild-type or the indicated myc-tagged human cortactin point-mutant constructs at 41°C (non-permissive temperature). TPM; triple point mutant consisting of Y-F mutations at positions 421, 470 and 486. After transfection, cells were cultured at 41°C, then shifted to 35°C (permissive temperature) for 2 h to promote v-Src activation. Recombinant cortactin proteins were assayed by immunoblotting with anti-cortactin-pY421, anti-cortactin-pS405, anti-myc, anti-cortactin, and anti-beta-actin antibodies. Note that the inability of cortactin to be phosphorylated on Y421 does not impact its ability to be phosphorylated on S405, nor does lack of S405 phosphorylation impact Y421 phosphorylation.

EGF treatment of UMSCC2 cells resulted in phosphorylation of cortactin S405/418 and cortactin pY421 (Figure 3A). A previous *in vitro* study evaluating the impact of cortactin phosphorylation on N-WASp activation determined that S405/418 phosphorylation by ERK1/2 enables the cortactin SH3 domain to stimulate N-WASp Arp2/3 activation, while Src phosphorylation downregulates N-WASp activity and counteracts the effects of S405/418 phosphorylation [15]. This proposed “on-off switch” postulates that cortactin serine and tyrosine phosphorylation are mutually exclusive events governing the ability of cortactin to regulate N-WASp activity and downstream actin reorganization [55]. Using the available antibodies reactive against cortactin pS405 and pY421, we sought to determine if these two different classes of phosphorylation events are interdependent in any manner. Cortactin depleted SYF fibroblasts (null for Src, Yes and Fyn kinases) were co-transfected with the temperature-sensitive vSrc construct *ts*La29-GFP [45] to activate the Src and ERK1/2 signaling pathways, along with constructs encoding wild-type cortactin or the following Myc-tagged cortactin mutants: Y421F, Y470F, Y486F, Y421/Y470/Y486F (TPM), S405A, S418A, S405/418A (Figure 4A). A W492K cortactin mutant was also included, as this mutant abolishes the ability of the cortactin SH3 domain to interact with corresponding SH3 binding proteins [56]. After shifting to 35°C for 2 h to activate *ts*La29-GFP, the serine and tyrosine cortactin mutants were analyzed for phosphorylation at Y421 and S405 by SDS-PAGE and Western blotting (Figure 4B). Mutations to S405 and S418 alone and in combination did not impact the ability of these constructs to be phosphorylated on Y421 (Figure 4B). Similarly, mutations to Y421, Y470, and Y486, alone and in combination (TYM) did not affect the ability of these constructs to be phosphorylated on S405. These data indicate that cortactin can be simultaneously phosphorylated at S405 and Y421 downstream of vSrc activation, suggesting in this system that phospho-regulation of cortactin SH3 domain function is not solely governed in vivo by the serine-tyrosine “on-off switch” mechanism proposed from previous in vitro experimentation [15], [55].

### S405/418 phosphorylation is required for efficient tumor cell motility and adhesion

**Figure 5:**
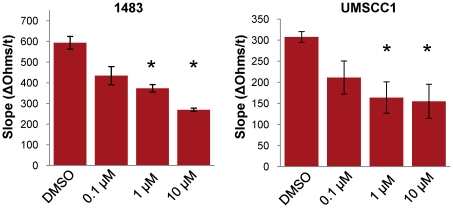
Targeted inhibition of MEK1/2 inhibits HNSCC cell motility. 1483 and UMSCC1 cells (5×10^5^) were starved for 24 h in the presence of vehicle (DMSO) or increasing concentrations of selumetinib as indicated. Cells were assayed for motility by electric substrate impedance sensing (ECIS) following stimulation with complete media containing the matched selumetinib concentration for 24 h. Data is displayed as slope values calculated from the linear part of ECIS tracings. Bars represent mean ± SE. *, p<0.05 compared to DMSO treated control cells.

**Figure 6:**
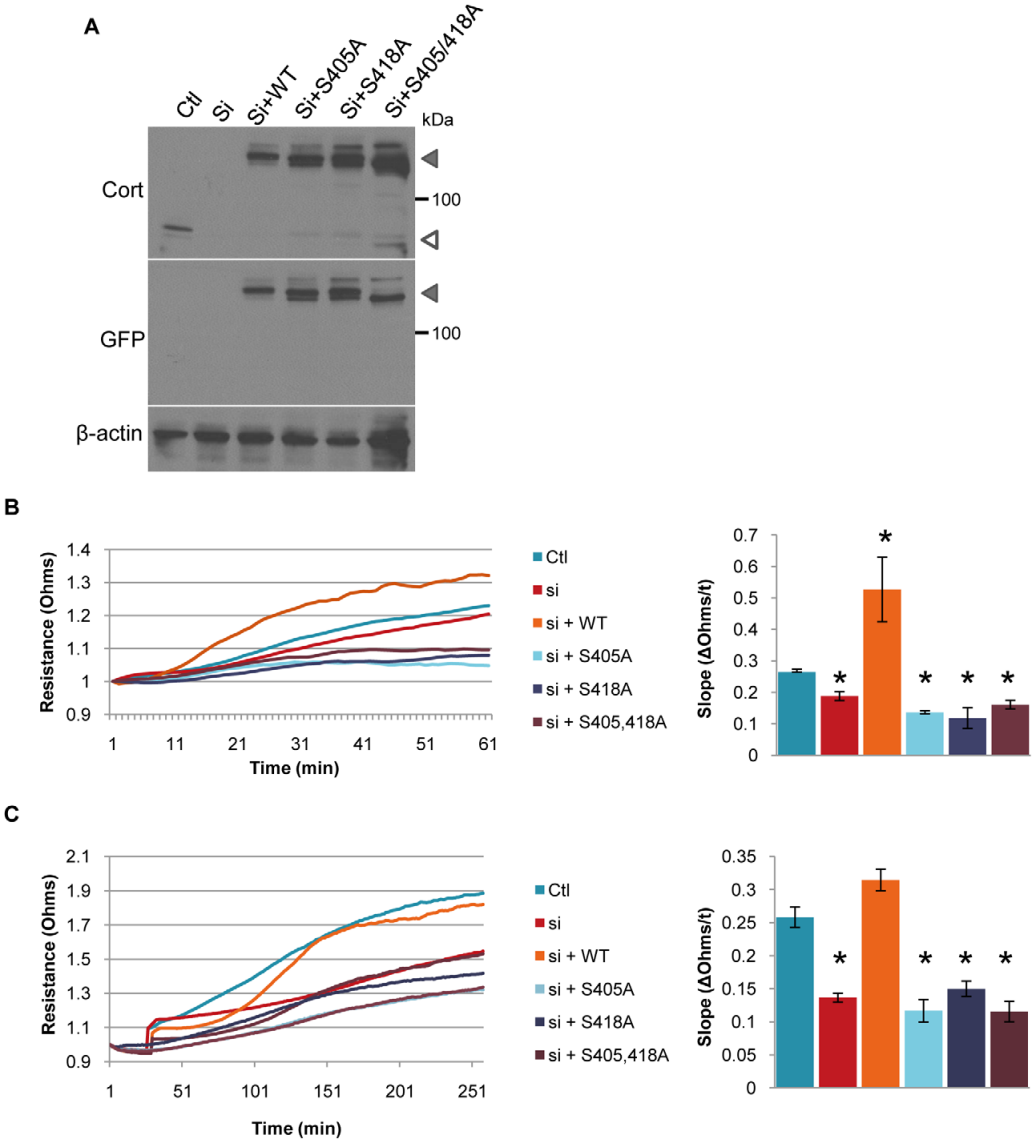
Cortactin phosphorylation at serine 405 and 418 regulates carcinoma cell migration and adhesion. (**A**) Expression of GFP-cortactin constructs in MTLn3 cells. MtLn3 cells were transfected with murine-specific cortactin siRNA (Si) for 48 h to silence endogenous cortactin expression. Cells were subsequently transfected with the indicated human GFP-tagged cortactin wild-type and the various Erk1/2 phosphorylation-null point mutant constructs. Following transfection, cell lysates were immunoblotted with anti-cortactin, anti-GFP and anti-beta-actin antibodies. Solid arrowheads indicate the position of GFP-tagged cortactin variants; open arrowheads denote the position of endogenous cortactin. (**B**) Serine 405 and 418 phosphorylation is required for efficient carcinoma cell motility. MTLn3 cells transfected as in (A) were analyzed for cell migration by ECIS. Cell impedance versus time plots for each transfected line are shown on the *left*; slope values calculated from the linear region of each plot are displayed on the *right*. (**C**) Carcinoma cell spreading requires phosphorylation of cortactin S405 and S418. Transfected MTLn3 cells were plated, with rates of spreading were monitored by ECIS tracings over time *left*. Slope values from the linear regions are shown on the right. Bars represent mean ± SE for three independent experiments. *, P<0.05 compared to control (ctl) cells.

To evaluate the role of cortactin S405/418 phosphorylation on carcinoma cell migration, 1483 and UMSCC1 cells were treated with selumetinib and assayed for effects on motility by ECIS (Figure 5). Selumetinib treatment impaired the motility of both cell types in a dose-dependent manner, corresponding to the observed decreases in S405/418 phosphorylation (Figure 2B). Since MEK inhibition likely impaired the phosphorylation of other proteins involved in motility in addition to cortactin, we directly assessed the impact of cortactin S405/418 phosphorylation on cell migration using phosphorylation-null cortactin expression constructs. MTLn3 rat mammary adneocarcinoma cells were initially transfected with a siRNA targeted against rodent cortactin, followed by transfection with GFP-tagged human wild-type (WT), S405A, S418A and S405/418A cortactin constructs. Cortactin siRNA reduced endogenous cortactin levels to >90%, having no impact on expression of the human GFP-labeled variants (Figure 6A). MTLn3 cells with cortactin knockdown (si) displayed a 29% reduction in motility compared to control (Ctl) (Figure 6B), similar to previous findings in MTLn3 cells and other cell types [18], [21], [57]. Expression of wild-type human GFP-cortactin (WT) led to a 2-fold increase in motility, presumably due to increased expression of this variant over endogenous (Ctl) levels (Figure 6A). Expression of S405A, S418A or S405,418A cortactin resulted in an 49% average decrease in cell migration for each cortactin mutant compared to Ctl, indicating that phosphorylation of S405 and S418 are vital in maintaining optimal carcinoma cell motility (Figure 6B). Since lamellipodia formation is required for detached cells to adhere to the ECM, we conducted ECIS assays to determine the effects of cortactin S405/418 phosphorylation on cell adhesion. MTLn3 cells lacking cortactin expression (si) exhibited a 50% decrease in cell adhesion compared to control (Ctl) cells. Expression of wild type (WT) GFP-cortactin restored adhesion to levels similar to Ctl, whereas expression of S405A, S418A or S405/418A cortactin mutants all reduced adhesion to levels 42–58% of Ctl, failing to restore adhesion to levels above cortactin si cells (Figure 6C). These results suggest that cortactin S405/418 phosphorylation contributes to carcinoma cell motility and adhesion, representing an important pro-migratory substrate targeted by the MEK-ERK1/2 pathway.

### Cortactin S405/418 phosphorylation is required for carcinoma cell lamellipodia persistence

**Figure 7:**
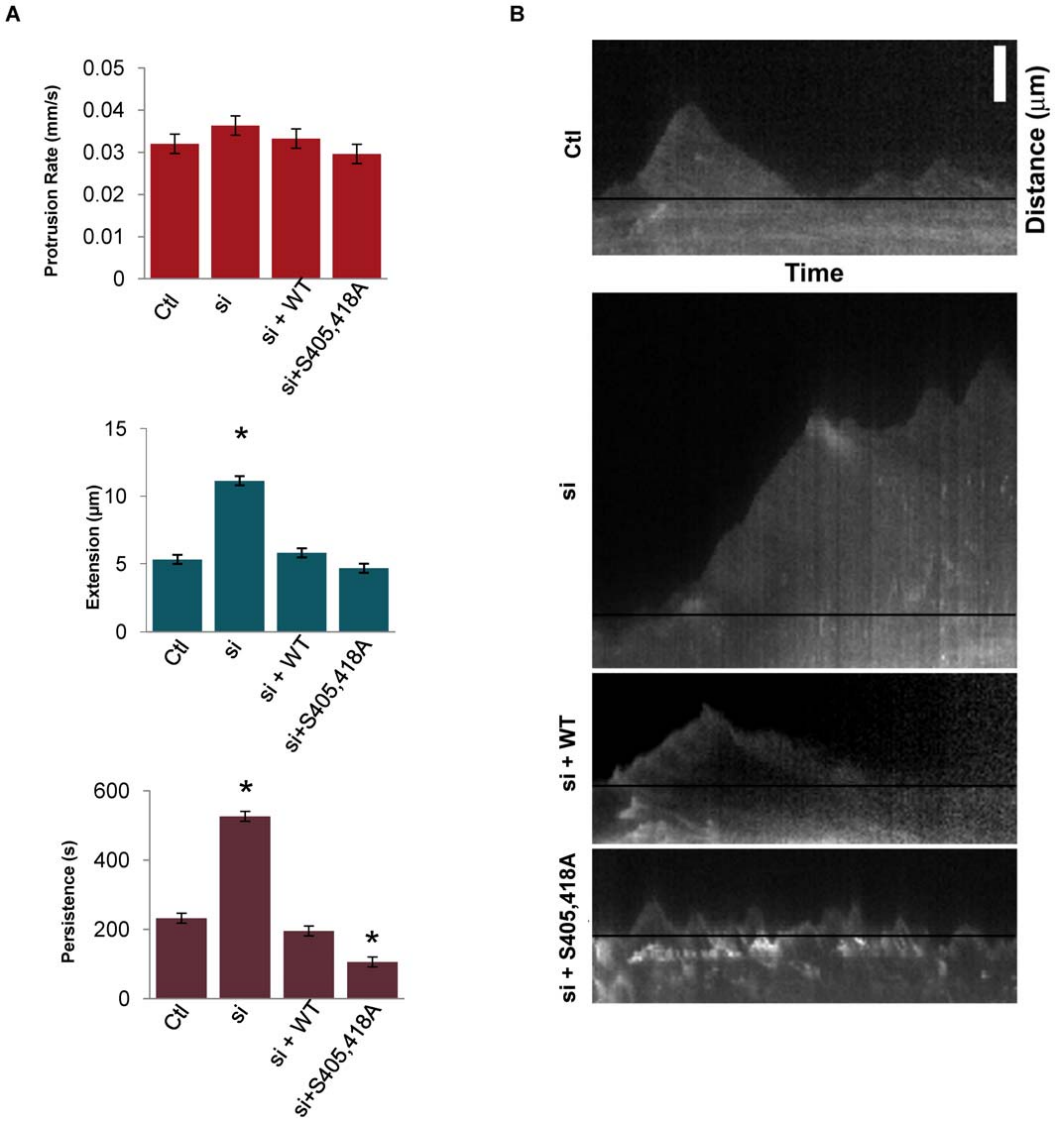
Cortactin phosphorylation at serine 405 and 418 is required for lamellipodia persistence. (**A**) Kymographic analysis of MTLn3 lamellipodia. Serum starved MTLn3 cells (Ctl) or cells transfected with the indicated cortactin siRNA and cortactin constructs were monitored for dominant lamellipodia formation by live cell imaging following EGF stimulation. Quantification of lamellipodia protrusion rates, length of extension, and time of lamellipodia persistence are shown for each experimental condition. ≥10 cells were analyzed for each group from ≥3 independent experiments. (**B**) Representative kymograms of each cell type. Kymograms were constructed from 1-pixel wide lines drawn from the initial leading edge and in the direction of the dominant lamellipodia. Cells were visualized by fluorescent microscopy using mCherry-beta-actin as the lamellipodia marker. Images were captured every five sec for a period of 15 min. Black lines denote the baseline position of the leading edge prior to EGF stimulation. Bar; 5 micrometers.

Given the localization of pS418 cortactin within lamellipodia (Figure 1B) and the effects of cortactin S405/418A expression on cell motility (Figure 6B), we evaluated the impact of cortactin S405/418 phosphorylation on lamellipodia dynamics using live-cell imaging and kymographic analysis. Serum-starved MTLn3 cells expressing mCherry-beta-actin and containing endogenous cortactin knockdown alone (si), rescued with human GFP- wild type cortactin (si+WT) or with GFP-cortactin S405/418A (si+S405,418) were stimulated with EGF for 15 min. Lamellipodia dynamics were monitored by time-lapse video microscopy (Video S1, Video S2, Video S3 and Video S4) and assayed by kymography (Figure 7A). EGF-stimulated MTLn3 cells produced an initial dominant lamellipodia that reached maximal extension between 1.5 and 3 min, and retracted to the point of origin between 5–7 min [58], [59]. Control MTLn3 cells containing mCherry-beta-actin displayed similar extension-retraction kinetics when assayed by kymography (Figure 7B and Video S1). While no differences were observed in lamellipodia protrusion rates in any of the assayed cellular conditions (Figure 7A), cortactin knockdown (si) increased lamellipodia extension by an average of 5.8 µm over the maximum extension length observed in control cells (Figure 7A and B). Lamellipodia formed in cortactin si cells failed to effectively retract, demonstrating a ∼2-fold increase in average lamellipodia persistence over control levels (Figure 7A and Video S2). These results are consistent with the observed increase in lamellipodia extension and persistence observed when MTLn3 cells contact EGF-coated bead matrices [21]. These effects are fully rescued to control levels upon expression of WT GFP-cortactin (si+WT; Figure 7A, Figure 7B and Video S3). Although expression of GFP-cortactin S405/418A in cortactin si cells did not impact EGF-induced lamellipodia extension, average lamellipodia persistence was reduced by 46%, from 195 sec in si+WT cells to 106 sec in si+405,418 cells (Figure 7A). The lamellipodia in si+405,418 cells displayed series of multiple short extensions and retractions, had enhanced ruffling and appeared more labile than control or si+WT cells (Figure 7B: Videos S1 and S3 compared to Video S4). These results suggest that S405/418 phosphorylation is critical in regulating lamellipodia actin dynamics responsible for proper protrusive behavior.

## Discussion

While the effects of cortactin phosphorylation at S405 and S418 by ERK1/2 have been studied at the biochemical and functional level in several systems [15], [26], [40], [41], the spatial and temporal evaluation of S405 and S418 phosphorylation have been hampered due to the lack of suitable reagents to directly study these sites in cellular and tissue contexts. Our development of anti-pS405 and anti-p418 cortactin antibodies allowed us to examine the localization and signaling pathways regulating these cortactin phosphorylation events. These antibodies, coupled with the use of phosphorylation-null mutant constructs, allowed us to validate and extend previous findings implicating these sites in the regulation of carcinoma cell motility and associated lamellipodia dynamics.

The localization of pS418 cortactin in carcinoma lamellipodia and invadopodia is consistent with the defined and emerging roles cortactin plays in regulating actin dynamics within these structures [10], [23]. To date, all studies designed to evaluate the cellular effects of pS405/418 phosphorylation have relied on the use of phosphorylation null or phosphomimetic (S405/418D) constructs. In pancreatic tumor cells, S405/418A and S405/418D both promote lamellipodia protrusion over control levels, whereas S405/418A inhibits and S405/418D promotes cell motility [41]. While the ability of S405/418A to promote lamellipodia protrusion in these studies is unclear, the remaining results are consistent with an activating role for S405/418 phosphorylation in lamellipodia dynamics and motility. Similar results were obtained in the analysis of S405/418 on invadopodia function, with S405/418A expression impairing and S405/418D promoting ECM degradation activity [26]. Phosphorylation of cortactin S418 within lamellipodia and invadopodia (Figure 1B) supports these results.

In HNSCC and several other tumor types, cortactin is present in the cytoplasm and is enriched at cell-cell junctions [48], [60], [61]. The localization of pS418 cortactin at regions of HNSCC cellular contact within tumors resembles the localization pattern of pY421 cortactin in this tumor type [62]. The staining pattern of cortactin and its tyrosine phosphorylated form is reminiscent of that found in two-dimensional epithelial monolayers, where cortactin has been shown to be essential for Arp2/3-mediated actin remodeling resultant from E-cadherin homoligation and subsequent Src activity [63], [64]. The presence of pS418 cortactin at these regions may suggest additional functional roles for cortactin in E-cadherin-mediated actin regulation within solid tumors.

Selumetinib inhibition of cortactin S405/S418 phosphorylation reinforces the MEK-ERK1/2 pathway as the main signaling route responsible for phosphorylating these cortactin sites in tumor cells [26], [40]. In addition to MEK, PAK1 has recently been shown to phosphorylate cortactin at S405/418 [42]. PAK1 is activated primarily by binding to active Cdc42 or Rac1 [65], which in turn binds and activates MEK to stimulate ERK1/2 activation [66]. Since MEK inhibition largely ablates S405/418 phosphorylation, the impact of PAK1 activity on S405/418 phosphorylation may be context dependent, with direct PAK1 phosphorylation of cortactin S405/418 regulating actin polymerization required for endosomal trafficking in contrast to impacting lamellipodia actin dynamics. While the current understanding regarding the interrelationship between PAK1 and MEK in governing cortactin S405/418 phosphorylation is incomplete, it is clear that the PAK-MEK-ERK1/2 signaling nexus impinges on cortactin to regulate actin dynamics involved in several membrane-based cellular processes.

Consistent with other reports [39], [40], we observed the MEK-dependent EGF-induced shifting of cortactin from the 80 kDa to 85 kDa form by Western blotting. The shift in cortactin *M*_r_ is not attributable to bulk addition of phosphate, since phosphatase treatment of cortactin immunoprecipitates from EGF-treated cells failed to reconvert the 85 kDa form to 80 kDa (data not shown). While the distinct 80 kDa and 85 kDa bands represent different post-translationally modified cortactin forms associated with pS405/418 phosphorylation, mutations at these sites have no effect on 80/85 kDa cortactin ratios, with the S405/418A mutant displaying a similar cortactin electrophoretic pattern to wild type cortactin (Figure 4). This suggests that S405/418 cortactin phosphorylation, while associated with the shift from 80 to 85 kDa, is not *necessary* for generation of the 85 kDa cortactin form. This is supported by the presence of 80 kDa and 85 kDa cortactin forms produced in kinase-free systems [27], [67] and by the existence of a single 85 kDa form when analyzed by urea denaturing SDS-PAGE [67].

The lack of detailed structural data for cortactin has hampered understanding conformational changes cortactin undertakes in response to post-translational modifying events. The existence of cortactin in a “closed” versus “open” form regulated by S405/418 phosphorylation has been proposed to explain the observed 80 to 85 kDa shift [15], [40]. These studies propose that the “closed” non-phosphorylated cortactin form assumes an autoinhibitory conformation where the SH3 domain binds back to an unidentified site within helical proline-rich (HP) domain to prevent binding to N-WASp and other SH3 ligands. Phosphorylation of S405/418 is proposed to liberate the SH3 domain, where it binds and stimulates N-WASp activation. This is supported by expression of cortactin S405/418D phosphomimetic forms in cells resulting in increased branched actin networks in actin tails associated with cytoplasmic vesicles [41]. Initial assessments of cortactin structure by electron microscopy revealed the protein as a rod shaped monomer 220 Å in length [68]. Recent biophysical studies utilizing chemical crosslinking and small angle x-ray scattering indicates that cortactin exists primarily in a globular form, with the carboxyl terminal HP and SH3 domains folding back onto the amino terminal actin binding region, supporting a “closed” conformation [69]. Additional evidence for an inhibitory function of the amino terminus can be inferred from the ability of the cortactin carboxyl terminal domain to promote N-WASP-dependent cell motility as effectively as wild type cortactin [16].

In the ERK-Src “switch” model, cortactin tyrosine phosphorylation by Src serves to downregulate N-WASp activity promoted by S405/418 phosphorylation [15]. This model therefore suggests that serine and tyrosine phosphorylation of cortactin function in a reciprocal manner to govern N-WASp activation [55]. Our data with site-specific phosphorylation antibodies indicates that S405/418 and Y421 can be co-phosphorylated, and analysis of point mutant cortactin constructs does not indicate a reciprocal influence between cortactin serine and tyrosine phosphorylation events. These data suggest that cortactin function is not exclusively regulated by a serine-tyrosine “switch” mechanism. While our data do not rule out scenarios where such a mechanism may be employed to some degree at the cellular level, they are consistent with biochemical and cellular studies demonstrating positive effects of cortactin tyrosine phosphorylation on N-WASp-mediated Arp2/3 activation [35], [37]. The ability of cortactin to be simultaneously phosphorylated at S405/418 and Y421/470/486 may therefore provide cells with the ability to fine-tune the level of N-WASp activation and subsequent actin remodeling in response to diverse upstream stimulatory input.

Inhibition of carcinoma cell motility by MEK blockade and S405/418A expression indicates that S405/418 cortactin phosphorylation is important in promoting and maintaining cell migration. While similar results were observed in wound healing assays [41], we extend these findings by evaluating the effects of pS405/418 on lamellipodia dynamics. The inability of MTLn3 cells expressing S405/418A cortactin to maintain EGF-stimulated dominant lamellipod persistence implies that the actin networks within these cells fail to maintain proper Arp2/3 nucleation, or are more labile following lamellipodia extension. While N-WASp activation and Arp2/3 mediated actin polymerization has been shown to be important in governing motility in multiple cell types [16], [41], WAVE2 and formin proteins, not N-WASp, are responsible for lamellipodia protrusion in MTLn3 cells [70]. These results would therefore rule out a role for direct N-WASp activation by pS405/418 cortactin in MTLn3 lamellipodia extension. In addition to N-WASp, the cortactin SH3 domain interacts with several other actin-regulatory proteins (reviewed in [10]). In particular, the cortactin SH3 domain activates faciogenital dysplasia protein 1 (FGD1) [71], [72], a potent activator of Cdc42 [44]. Cdc42 activity is required for localization of WAVE2 and its activator IRSp53 to the cell membrane, where it mediates lamellipodia extension [73]. FGD1 also activates the MEK-ERK1/2 pathway [44], providing the potential of a positive feedback loop in stimulating cortactin S405/418 phosphorylation through continuous cortactin SH3-mediated FGD1 activity. Whether such an FGD1-based regulatory circuit or other modes of potential pS405/418 cortactin regulation of WAVE2 exist in MTLn3 cells remains to be evaluated.

Previous studies on lamellipodia dynamics in other cells types indicate that cortactin removal decreases lamellipodia persistence, which can be rescued by re-expression of a cortactin amino terminal fragment lacking the carboxyl terminal region [18] and therefore eliminating contributions from pS405/418 in this system. These results differ from our work in MTLn3 cells, where cortactin removal results in enhanced persistence. These differences may be due to a combination of different cell types, chemotatic cues, and analysis of the dominant, initial lamellipodia versus steady-state lamellipodia dynamics [10]. Interestingly, inhibition of ERK1/2 signaling during macrophage lamellipodia extension results in decreased lamellipodia stability, with similar kymograph profiles to EGF-stimulated MTLn3 cells with S405/418A expression [74]. These studies provide supporting evidence for our observations.

## Supporting Information

Figure S1 EGFR activation status in 1483 cells in response to selumetinib or saracattinib treatment. 1483 cells were treated with vehicle (DMSO), selumetinib (A), or saracatinib (B) for 16 h in serum free media. Cells were stimulated with 100 nanograms/ml EGF for 20 min, lysed and analyzed by Western blotting with anti-EGFR-pY1068, anti-EGFR, anti-pErk1/2, Erk1/2, anti-Src-pY418, and anti-Src antibodies as indicated.
(0.23 MB TIF)

Video S1 EGF-mediated extension of MTLn3 cells. Time lapse video microscopy of non-transfected mCherry-beta-actin transfected MTLn3 cells stimulated with 100 nanograms/ml EGF. Cells were visualized by swept-field fluorescence microscopy.
(0.30 MB MOV)

Video S2 Cortactin depletion enhances lamellipodia persistence MTLn3 cells stimulated with soluble EGF. MTLn3 cells were transfected with siRNA targeting cortactin. 48 h after transfection, cells were transfected with mCherry-beta-actin and stimulated with EGF. Cells were visualized by swept-field fluorescence microscopy.
(0.15 MB MOV)

Video S3 Expression of wild-type cortactin rescues siRNA-mediated enhancement of lamellipodia persistence. MTLn3 cells were transfected with siRNA targeting cortactin were co-transfected after 48 h with mCherry-beta-actin and GFP-tagged wild type cortactin. Cells were visualized by swept-field fluorescence microscopy. Red channel, mCherry; green channel; GFP fluorescence.
(4.43 MB MOV)

Video S4 Phosphorylation of cortactin S405/418 is required for lamellipodia persistence. MTLn3 cells transfected with siRNA targeting cortactin were co-transfected after 48 h with mCherry-beta-actin and GFP-labeled S405/418A cortactin. Cells were visualized by swept-field fluorescence microscopy. Red channel, mCherry; green channel; GFP fluorescence.
(7.00 MB MOV)
