# Supplementary figures and images for: Cortactin Phosphorylated by ERK1/2 Localizes to Sites of Dynamic Actin Regulation and Is Required for Carcinoma Lamellipodia Persistence
Source: PLoS One. 2010 Nov 4;5(11):e13847. doi: 10.1371/journal.pone.0013847 (PMC2973953; doi:10.1371/journal.pone.0013847)

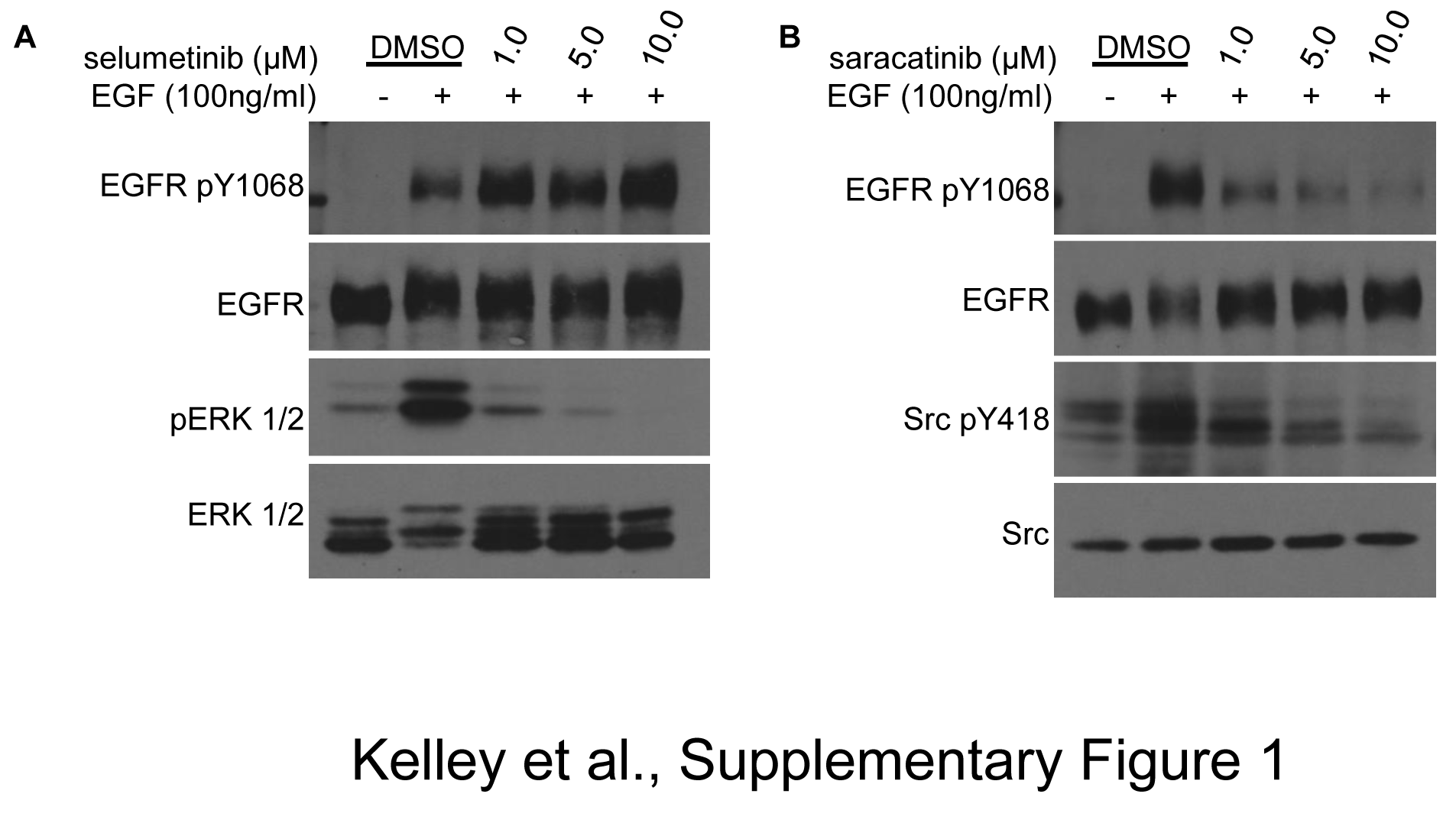

Supplement: Figure S1 — EGFR activation status in 1483 cells in response to selumetinib or saracattinib treatment. 1483 cells were treated with vehicle (DMSO), selumetinib (A), or saracatinib (B) for 16 h in serum free media. Cells were stimulated with 100 nanograms/ml EGF for 20 min, lysed and analyzed by Western blotting with anti-EGFR-pY1068, anti-EGFR, anti-pErk1/2, Erk1/2, anti-Src-pY418, and anti-Src antibodies as indicated. (0.23 MB TIF) [file pone.0013847.s001.tif]
